# Supplementary material for: Dynamic control of adipose tissue development and adult tissue homeostasis by platelet-derived growth factor receptor alpha
Source: eLife. 2020 Jun 19;9:e56189. doi: 10.7554/eLife.56189 (PMC7338051; doi:10.7554/eLife.56189)
Supplement: Supplementary file 1. [file elife-56189-supp1.docx]

Table S1

Primer sequences used

| Gene | Forward | Reverse |
| --- | --- | --- |
| *Fabp4* | AAGGTGAAGAGCATCATAACCCT | TCACGCCTTTCATAACACATTCC |
| *Pparg2* | TCGCTGATGCACTGCCTATG | GAGAGGTCCACAGAGCTGATT |
| *Plin1* | GGGACCTGTGAGTGCTTCC | GTATTGAAGAGCCGGGATCTTTT |
| *Col1a1* | AGATGATGGGGAAGCTGGCAA | AAGCCTCGGTGTCCCTTCATT |
| *Col3a1* | ATTCTGCCACCCCGAACTCAA | ACAGTCATGGGGCTGGCATTT |
| *Col6a1* | CTGCTGCTACAAGCCTGCT | CCCCATAAGGTTTCAGCCTCA |
| *Adipoq* | GCAGGCATCCCAGGACATC | GCGATACATATAAGCGGCTTCT |
| *Pnpla2* | GTGAAGCAGGTGCCAACATTATTG | AAACACGAGTCAGGGAGATGCC |
| *Ddr2* | ATCACAGCCTCAAGTCAGTGG | TTCAGGTCATCGGGTTGCAC |
| *Lep* | AAGACCATTGTCACCAGGATCAA | GGATACCGACTGCGTGTGTG |
| *Pdgfra* | TCCATGCTAGACTCAGAAGTCA | TCCCGGTGGACACAATTTTTC |
| *Rn18s* | GTAACCCGTTGAACCCCATT | CCATCCAATCGGTAGTAGCG |
